# Exploring Online and In-Store Purchase Willingness: Associations With the Big Five Personality Traits, Trust, and Need for Touch

**DOI:** 10.3389/fpsyg.2022.808500

**Published:** 2022-04-04

**Authors:** Anna Hermes, Cornelia Sindermann, Christian Montag, René Riedl

**Affiliations:** ^1^Institute of Business Informatics-Information Engineering, Johannes Kepler University Linz, Linz, Austria; ^2^Department of Molecular Psychology, Institute of Psychology and Education, Ulm University, Ulm, Germany; ^3^Digital Business, University of Applied Sciences Upper Austria, Steyr, Austria

**Keywords:** consumer personality, Big Five, trust, need for touch, willingness to purchase, online shopping, in-store shopping, cross-channel shopping

## Abstract

Nowadays, customers can utilize both online and in-store retail channels. Consequently, it is crucial for retailers to understand the possible drivers of retail channel selection, including customers’ personalities, degrees of trust, and product touch preferences. Unfortunately, current omnichannel research only scarcely addresses the effects of personality, trust, and desire to touch a product before purchasing it on willingness to purchase and how those effects vary between online and in-store shopping. Thus, we conducted an exploratory study. Our analysis of survey data (*N* = 1,208)—which controls for respondents’ age, gender, and education—reveals that across both the willingness to purchase in-store and online, a higher level of e-vendor trust is a significant, positive predictor. However, we also identify several channel-related differences, including that Trust Propensity, as well as the Big Five traits of Extraversion, Agreeableness, and Conscientiousness are significantly positively related to in-store, but not online, purchase willingness. We also find that Instrumental Need for Touch (defined as goal-motivated touch of a product) is positively related to in-store, but negatively related to online, purchase willingness. Finally, we highlight opportunities for future research and discuss how retail managers might enhance customer experiences in their physical and online stores.

## Introduction

For retailers who wish to remain competitive, the rise of online shopping during the COVID-19 pandemic (Scott et al., 2020; Sheth, 2020) has made the provision of omnichannel retailing—the seamless integration of retail channels—and personalized customer experiences more important than ever (Lemon and Verhoef, 2016; Kannan and Li, 2017; Manser Payne et al., 2017; Piroth et al., 2020b). To develop these targeted, omnichannel marketing strategies, retail managers must understand the effects of consumers’ underlying cognitive, affective, and behavioral tendencies (Puccinelli et al., 2009; Verhoef et al., 2009; Grewal and Roggeveen, 2020). Against this background, we answer recent and explicit calls to explore the effects of individual factors, such as personality, on purchase willingness or intention (Lemon and Verhoef, 2016; Grewal and Roggeveen, 2020; Handarkho, 2020; Piroth et al., 2020a; Hermes and Riedl, 2021b).

While some studies have already explored the effects of established personality theories, such as the Five-Factor Model and the Big Five traits, on online purchasing intentions (e.g., Bosnjak et al., 2007; McElroy et al., 2007), there is still little research on the impact of Big Five traits on willingness to purchase in-store, and studies examining the traits’ impact on both in-store willingness to purchase and online willingness to purchase are even scarcer (Hermes and Riedl, 2021b). To the best of our knowledge, only one study has investigated differences between online and in-store behavior regarding the direct effects of any Big Five trait: Breazeale and Lueg (2011) examined the effects of Extraversion (among other factors) on retail channel preference. However, none of the remaining Big Five traits were included in Breazeale and Lueg (2011) research. In short, despite that customers may choose to purchase products either online or in-store, the scientific literature has thus far overlooked the potential role of the Big Five with respect to these impactful channel-selection decisions.

Because online shopping is considered risky (Peck et al., 2013; Bezes, 2016), the importance of a customer’s Trust Propensity (TP) and E-Vendor Trust (EVT) has been acknowledged in prior literature, particularly in the field of Information Systems (McKnight et al., 1998; Gefen, 2000). TP refers to the general, i.e., not only in specific situations, tendency to have faith in humanity and to show a general willingness to rely on others, whereas EVT refers to a customer’s trust in a specific e-vendor (McKnight et al., 1998; Gefen, 2000). Researchers have also established that trust can flow between channels—that is, from offline trust (namely trust towards the company, products, and sales personnel) to the online shop (called “trust transfer”; see Jeon et al., 2021) or from an online website to an “offline site” (e.g., an e-vendor’s link to information about a physical store location, Lee et al., 2014). Despite this, TP and EVT have only been researched, to the best of our knowledge, in online shopping contexts (e.g., Gefen, 2000; Gefen et al., 2003b). Given the importance of trust in marketing literature at large (Morgan and Hunt, 1994; Heidarian, 2019), we aim to explore whether these two trust variables, TP and EVT, also have effects in in-store environments. Accordingly, when *trust* appears below, it refers to both constructs, jointly represented.

Another integral part of the customer journey is physical engagement with (that is, the touching of) a product (Roggeveen et al., 2020), which helps consumers to form impressions of that product (Ranaweera et al., 2021), but individuals differ in their Need for Touch (NFT). While affect and cognition play important roles in the customer experience (Gentile et al., 2007; Puccinelli et al., 2009; Hermes and Riedl, 2020a,b), NFT can also be motivated by enjoyment and pleasure (affective side, so-called Autotelic NFT) or to receive product information, such as a product’s weight or quality (based on analytic thoughts, goal-driven, so-called Instrumental NFT, Peck and Childers, 2003a). Online consumption lacks the element of touch (Peck and Childers, 2003a), and accordingly, online customers displayed lower levels of NFT (San-Martín et al. (2017); however, Duarte and Costa e Silva (2020) did not confirm this finding). Customers high in NFT, meanwhile, are willing to pay higher prices in-store and have stronger concerns with product quality online (Kühn et al., 2020). Yet, to the best of our knowledge, no study so far has explored personality constructs, such as the Big Five, alongside trust and the shopping-specific construct of NFT across both online and in-store purchasing contexts.

To respond to this research gap, we conducted an online survey study using the consumer data of a fashion retailer from Central Europe. A large sample of this retailer’s customers (*N* = 1,208) were surveyed to examine the associations of the Big Five, trust, and NFT with willingness to purchase in-store and online. Since various studies have demonstrated that age (e.g., Hult et al., 2019; Dorie and Loranger, 2020), gender (e.g., Seock and Bailey, 2008; Hult et al., 2019; Mann and Liu-Thompkins, 2019), and education (e.g., Hult et al., 2019) can influence customers’ purchase behavior and retail channel-selection behavior, we control for these three customer demographics so as to rule out their effects. The novel contributions of this exploratory study are as follows:

First, notwithstanding the current rise of e-commerce by the COVID-19 pandemic, it is likely that some of these online-purchasing habits may to some degree be reversed back to in-store shopping in a post-pandemic world (Sheth, 2020; Unnikrishnan and Figliozzi, 2021). Consequently, research on the drivers of purchase-channel decisions is becoming increasingly important. However, studies that explore the associations between multiple psychological factors (such as the Big Five, trust, and preferences like NFT) and customers’ willingness to purchase products online and in-store remain scarce. The present research attempts to fill this void.

Second, the present work contributes to the theory of several domains of research. The research described herein pursues the three following research objectives: (a) to determine the relationship between Big Five traits and willingness to purchase online and in-store, (b) to determine the relationship between trust and willingness to purchase online and in-store, and (c) to determine the relationship between NFT and willingness to purchase online and in-store. Therefore, our findings related to Big Five traits, trust, and NFT supplement existing literature in the field of consumer psychology. Furthermore, this study adds to the existing knowledge in customer-behavior literature in general.

Finally, this work offers recommendations as to how retail managers can optimize customer experiences in accordance with our findings on the effects of the Big Five, trust, and NFT across both online and in-store retailing.

The remainder of this paper is structured as follows: section “Theoretical Background” discusses the theoretical background of the study; section “Materials and Methods” describes its methods and materials; section “Results” presents our results; and section “General Discussion” opens a discussion of the implications of our work in both research and practice, as well as detailing limitations of the study and future research opportunities.

## Theoretical Background

### Research Objective 1: The Big Five and Willingness to Purchase Online and In-Store

A consumer’s personality—defined as their set of “stable individual differences in cognitive, emotional and motivational aspects of mental states that result in stable behavioral action (especially emotional) tendencies” (Montag and Panksepp, 2017, p. 1)—can influence his or her purchase decisions and customer experience (see, e.g., Verhoef et al., 2009). According to the well-known Five-Factor model, personality can be assessed by measuring five traits: (a) Extraversion, (b) Agreeableness, (c) Conscientiousness, (d) Neuroticism (whose opposite is Emotional Stability), and (e) Openness to Experience (hereafter Openness). For a brief historical overview of the discovery of the Big Five, please see Montag and Elhai (2019).

According to Costa and McCrae (1992), each Big Five trait corresponds to a set of unique characteristics. People scoring high in Neuroticism, for example, tend to experience higher emotional distress, while those scoring high in Extraversion are inclined to sociability, activity, and positive emotions (e.g., joy). Those who score high in Openness are considered to be imaginative and to possess strong aesthetic sensibility. Individuals with high Agreeableness scores tend to be more trusting and empathic (Melchers et al., 2016). Finally, people scoring high in Conscientiousness are well organized and disposed toward structure and order (Costa and McCrae, 1992).

The associations of the Big Five with online shopping behavior and intentions have been documented in the past. For example, McElroy et al. (2007) found that in online shopping, of all Big Five traits, only Neuroticism showed a significant, positive association with e-buying and e-selling. Meanwhile, Bosnjak et al. (2007) found significant relationships between online purchase intentions and Openness, Agreeableness, and Neuroticism (positive, negative, and negative relationships, respectively). Later, Mohamed et al. (2014) found that while Extraversion was positively correlated with customers’ online shopping continuance intentions, Emotional Stability (as the opposite pole of Neuroticism) was not associated with online shopping continuance intentions. Another team of scholars, Moslehpour et al. (2018), reported that Conscientiousness had a significant, positive correlation with online purchase intentions and that relationship was mediated by perceived ease of use and perceived usefulness; Openness was found to have no impact on online purchase intentions. Interestingly, in a study by Piroth et al. (2020a), no Big Five trait predicted customer attitudes toward online grocery shopping, and Lixăndroiu et al. (2021) also did not find direct effects of the Big Five on online buying intention.

While no study, to the best of our knowledge, has examined the relationships between Big Five traits and willingness to purchase in-store (excepting that of Breazeale and Lueg, 2011, who considered Extraversion within a mall-shopping context), the literature does describe the Big Five’s impacts on in-store shopping and its motivations in general. Coshall and Potter (1986) were among the first to consider the impact of the Big Five on shopping-center selection. In their study, they found that consumers with high levels of Emotional Stability (as the opposite pole of Neuroticism) were likely to visit fewer shopping centers known to them in comparison to consumers reporting high levels of Neuroticism. Further, consumers with high levels of Extraversion reported a greater tendency to use more of the shopping centers known to them in comparison to consumers with high levels of Introversion. Mooradian and Olver (1996) concluded that the Big Five affected shopping motives; among other findings, they reported that customers high in Neuroticism shopped for mood-management purposes (e.g., self-gratification), while customers high in Extraversion sought a wide range of social shopping experiences. The two researchers also described sensory stimulation and learning about trends as important to higher-Openness customers, while highly agreeable customers were seen to enjoy finding a bargain but not to enjoy bargaining itself. Lastly, Conscientiousness in customers was positively associated with enjoying bargains and learning about new trends but negatively related to shopping for self-gratification (Mooradian and Olver, 1996). Guido (2006) also surveyed shopping-center customers and found relationships between Big Five traits and the hedonic (e.g., shopping for enjoyment) and utilitarian (e.g., functional and task-related shopping) forms of motivation. Specifically, hedonic shopping motivations were positively associated with Openness, Extraversion, and Agreeableness, while utilitarian shopping motivations were positively associated with Emotional Stability (as the opposite pole of Neuroticism) and Conscientiousness (Guido, 2006). Finally, the aforementioned study by Breazeale and Lueg (2011) considered the effects of Extraversion on both online and mall shopping behavior among teenagers in the United States; individuals scoring higher in Extraversion were more likely to shop at malls and less likely to shop online.

In summary, the existing literature points to associations between Big Five traits and online shopping behavior. However, findings regarding exactly which Big Five traits are related to these kind of behaviors, as well as the direction of and severity of those associations, are as of yet inconclusive (see, e.g., the concurrent studies of Bosnjak et al. (2007) who found a negative; and McElroy et al. (2007) who found a positive association between Neuroticism and online-purchasing behavior). In short, the true nature of the Big Five traits’ relationships with online shopping remains undiscovered. Furthermore, some researchers, such as Piroth et al. (2020a) and Lixăndroiu et al. (2021), have found no direct relationships at all between Big Five traits and online shopping behavior (Piroth et al. (2020a) considered attitudes toward the adoption of online grocery shopping; and Lixăndroiu et al. (2021) researched online buying intentions). As for in-store shopping, prior research has shown that Big Five traits can affect related motivations, but studies on the relations of Big Five traits with willingness to purchase in-store remain scarce (Hermes and Riedl, 2021b). Finally, despite the recent trend toward omnichannel retailing, the majority of published research has considered either online shopping or in-store shopping, but not both (Hermes and Riedl, 2021b). Thus, we add to the literature by exploring the effects of all Big Five traits on in-store and online willingness to purchase, and we expected the following:

Proposition 1: The Big Five traits explain incremental variance in customers’ willingness to purchase online and willingness to purchase in-store after age, gender, and education have been considered in the analysis.

### Research Objective 2: Trust and Willingness to Purchase Online and In-Store

The importance of trust in the Information Systems discipline at large—and, hence, in the online shopping context in particular—has been widely studied (see, e.g., Gefen, 2000; McKnight et al., 2002a; Gefen et al., 2008; Riedl et al., 2010; Söllner et al., 2016). Given the vast body of literature on the subject, we refer below only to those studies that specifically considered the direct effects of TP (also called “disposition to trust” or “trust disposition”) and EVT (or “trust in the e-vendor”) on online or in-store purchase intentions. Bianchi and Andrews (2012) anticipated a positive correlation between TP and customer intention to shop online, but discovered a significant, negative relationship instead. In contrast, McKnight et al. (2002a) found no association between TP and trust-related online behavior (including making purchases). EVT, another important construct in the domain of online purchasing (Gefen, 2000), has also been the subject of various studies, which identified positive relationships between EVT and the use of an e-vendor’s website (Gefen et al., 2003b; Kim, 2008), the intention to shop online (Gefen and Straub, 2003; Gefen et al., 2003a; Kim, 2014), and the willingness to commit in a long-term relationship with the retailer (Cho, 2006). In omnichannel contexts, retailers engage with their customers through various channels, and to the best of our knowledge, no study has examined the influence of both TP and EVT on customers’ in-store purchase willingness (Lee et al. (2014) considers a trust transfer from an online website to an “offline site”, such as an e-vendor’s link to information about a physical store location, but does not consider TP). Given this research gap, and in light of previous studies of offline-to-online (Kuan and Bock, 2007; Bock et al., 2012; Lee et al., 2014) and online-to-offline (Lee et al., 2014) trust transfer, we explore the effect of TP and EVT (together represented by “trust”) on in-store and online willingness to purchase, and we expected the following:

Proposition 2: Trust explains incremental variance in customers’ willingness to purchase online and willingness to purchase in-store after age, gender, education, and the Big Five have been considered in the analysis.

### Research Objective 3: Need for Touch and Willingness to Purchase Online and In-Store

Various researchers have explored the link between customers’ varying levels of NFT and their shopping behavior, though it should be noted that not all of them considered Autotelic NFT and Instrumental NFT separately. Evidence indicates that customers with higher levels of NFT in general (Cho and Workman, 2011), and of both Autotelic NFT and Instrumental NFT in particular (Workman and Cho, 2013), tend to prefer shopping channels that allow for touch. Correspondingly, customers with higher Autotelic NFT were more likely to purchase impulsively at a supermarket than were customers with lower Autotelic NFT (Peck and Childers, 2006). Customers scoring higher in NFT were also willing to pay higher prices for groceries at local stores (Kühn et al., 2020). When San-Martín et al. (2017) examined NFT they found that the higher a customer’s e-commerce orientation, the lower his/her NFT; however, Duarte and e Silva (2020) were unable to establish a relationship between level of NFT and propensity to purchase online. Evidence indicates that customers scoring higher in NFT perceive products as lower quality when viewed online, as opposed to in-store (San-Martín et al., 2017; Kühn et al., 2020), and NFT is positively associated with webrooming intentions (searching online and buying in-store; Aw, 2020; Aw et al., 2021; Shankar and Jain, 2021). Altogether, the studies tend to suggest that a customer’s level of NFT can significantly impact their online and in-store purchase behavior. Accordingly, we expected the following:

Proposition 3: Need for Touch explains incremental variance in customers’ willingness to purchase online and willingness to purchase in-store after age, gender, education, the Big Five, and trust have been considered in the analysis.

Table 1 summarizes related work examining the impact of the Big Five, trust, and NFT on online and in-store purchase willingness.

**Table 1:** Summary of related work examining the relations of the Big Five, trust, and NFT with online and in-store purchase willingness.

Source	Method	Sample size	Region	Context		Findings
				Online	In-Store	
**Big Five**						
Bosnjak et al., 2007	Survey	808	Croatia	x		Openness was positively associated with online purchase intention. Agreeableness, Neuroticism were negatively associated with online purchase intention. Conscientiousness, Extraversion were not associated with online purchase intention.
Breazeale and Lueg, 2011	Survey	583	United States	x	x	US teens high in Extraversion showed high mall- and low internet-shopping behavior.
Coshall and Potter, 1986	Survey	211	United Kingdom		x	Consumers with high levels of Emotional Stability were likely to visit fewer shopping centers known to them in comparison to consumers scoring higher in Neuroticism. Consumers with high levels of Extraversion reported a greater tendency to use more of the shopping centers known to them in comparison to more introverted consumers.
Guido, 2006	Survey	600	Italy		x	Openness, Extraversion, Agreeableness were positively linked to hedonic shopping values. Emotional Stability, Conscientiousness were positively linked to utilitarian shopping values.
Lixăndroiu et al., 2021	Quasi-experiment, Survey	121	N/A	x		The Big Five were not directly associated with online buying intentions.
McElroy et al., 2007	Survey	153	N/A	x		Openness, Agreeableness, Conscientiousness, Extraversion were not associated with e-buying or e-selling. Neuroticism was positively associated with e-buying and e-selling.
Mohamed et al., 2014	Survey	197	Malaysia	x		Extraversion was positively associated with online shopping continuance intention. Emotional Stability was not associated with online shopping continuance intention.
Mooradian and Olver, 1996	Survey	211	N/A		x	Agreeableness was, among others, associated negatively with bargaining and positively with enjoying bargains. Conscientious customers enjoyed bargains and learning about new trends but did not shop in-store for self-gratification, which was inversely related to Conscientiousness. Extraversion was positively associated with social shopping motives, such as talking with others. Neuroticism was positively associated with in-store shopping for physical activity, self-gratification, and sensory stimulation, but was inversely related to bargaining. Openness was positively associated with sensory stimulation and learning about new trends.
Moslehpour et al., 2018	Survey	316	Taiwan	x		Openness was not associated with online purchase intention. Conscientiousness was indirectly (through perceived ease of use, perceived usefulness) and positively associated with online purchase intention.
Piroth et al., 2020a	Survey	678	Germany	x		The Big Five were not associated with attitude toward buying groceries online.
Wang et al., 2006	Survey	473	Taiwan	x		Openness was indirectly (through attitude toward online shopping) positively associated with online purchase intention.
**Trust Propensity**						
Bianchi and Andrews, 2012	Survey	176	Chile	x		Consumer propensity to trust was negatively associated with intention to continue making online purchases.
McKnight et al., 2002a	Experiment, Survey	1,403	N/A	x		Disposition to trust was not associated with trust-related intentions toward an e-vendor (e.g., intending to make a purchase).
**E-Vendor Trust**						
Cho, 2006	Survey	881	N/A	x		EVT was positively associated with a customer’s willingness to commit to a retailer long-term.
Gefen and Straub, 2003	Experiment, Survey	161	United States	x		EVT was positively associated with online purchase intention.
Gefen et al., 2003a	Experiment, Survey	317	N/A	x		EVT was positively associated with online purchase intention; this effect was stronger for potential customers (as opposed to repeat i.e., experienced, customers).
Gefen et al., 2003b	Survey	213	United States	x		EVT was positively associated with intention to use a business-to-consumer website.
Kim, 2008	Survey	445	United States, South Korea	x		EVT was positively associated with willingness to use an e-vendor’s website.
Kim, 2014	Survey	249	United States	x		EVT was positively associated with customer intention to repurchase from an e-vendor.
**Need for Touch**						
Aw et al., 2021	Survey	280	Malaysia	x	x	NFT was positively associated with webrooming intention.
Aw, 2020	Survey	210	Malaysia	x	x	NFT motivated webrooming intention.
Cho and Workman, 2011	Survey	277	United States		x	Participants with high levels of NFT preferred shopping channels that allowed for touch.
Duarte and Costa e Silva, 2020	Survey	295	Portugal, China	x		Consumer NFT was not associated with consumer propensity to purchase online.
Kühn et al., 2020	Experiment, Survey	199, 181, 104	Germany	x	x	Customers scoring higher in NFT were more likely to accept higher prices for groceries sold in local stores. High NFT was linked to stronger concerns with quality and less positive affective responses to groceries offered online.
Peck and Childers, 2006	Survey	170	United States		x	In-store customers with higher Autotelic NFT were more likely to purchase impulsively than those with lower Autotelic NFT.
San-Martín et al., 2017	Survey	540	Spain	x	x	The greater a participant’s e-commerce orientation, the lower his/her NFT. Participant’s NFT decreases his/her perceived product quality stronger in online than in in-store shopping contexts.
Shankar and Jain, 2021	Survey	374	India	x	x	NFT was positively associated with webrooming intentions for luxury goods.
Workman and Cho, 2013	Survey	263	South Korea		x	Participants with high levels of Autotelic and Instrumental NFT reported stronger preferences for shopping channels that allowed for touch.

*EVT, E-Vendor Trust; NFT, Need for Touch; and N/A, Not Applicable/Not Provided*.

## Materials and Methods

### Open Practices, Procedure, Ethics, and Participants

Convenience sampling was used for data collection. This preregistered study^1^ was conducted online using LimeSurvey.^2^ Participants in the study were recruited *via* email solicitation by a fashion retailer from Central Europe. Customers who had previously given permission to receive marketing and advertising emails (e.g., who registered to receive the retailer’s newsletter or participated in the retailer’s loyalty card program) were contacted. The retailer emailed a total of 77,024 customers over a period of 4 weeks, beginning in mid-November 2020. To encourage participation, respondents were able to win one of five €100 coupons (provided by the retailer). Participation was open to customers who satisfied two criteria: (a) possessing the ability to speak German and (b) having shopped at the retailer’s online or physical store between June 2019 and October 2020. Limiting the time frame to this period allowed us to avoid bias that may otherwise have been introduced to the data by customers whose online shopping experiences took place on an outdated e-commerce website (the retailer’s website underwent a relaunch in May 2019). The first page of the survey listed both requirements and stated that only participants who fulfilled both criteria were eligible to participate. Austrian law governing university research exempts pure survey studies from the requirement for ethical approval. Informed consent was electronically obtained from all subjects prior to their participation in the study. Participants were able to participate in the survey until the end of December 2020.

A total of *N* = 1,229 individuals participated in the online survey (response rate approximately 1.6%). This rate is lower than response rates from other personality survey studies (e.g., Blair et al. (2018) who studied students’ personality characteristics and their impact on engagement leader development and reported a response rate of 29%) or other consumer behavior studies (e.g., Echchakoui (2017) who considered the effect of salesperson personality on sales performance with a response rate of 18%). However, considering that this study included actual retail customers, it was expected that this study might result in a lower response rate. Also, the length of the survey may have affected the rate of completed responses. After the data-cleaning procedure, the final sample size was *N* = 1,208 study participants (*n* = 596 men, *n* = 609 women, *n* = 3 third gender or gender-diverse; to review the data-cleaning procedure, please refer to Appendix A1). The mean age of the sample was 46.84 years, with a standard deviation of 12.21 years. Most participants reported vocational training (*n* = 375) as their highest educational attainment, followed by other vocational schools (*n* = 234), university degree including master’s, Magister, diploma degrees (*n* = 178), specialist course or college similar in character to universities (*n* = 108), high school (*n* = 102), university degree including bachelor’s and baccalaureate degrees (*n* = 86), other schooling (*n* = 51), general schooling/secondary modern school (*n* = 39), and university degree including Doctorate and PhD degrees (*n* = 35).

### Measures

#### Demographics

Participants answered demographic questions, reporting their age, gender, and highest level of education.

#### Personality

The German version of the Big Five Inventory (BFI) was used to assess personality (John et al., 1991; Rammstedt and Danner, 2017). This questionnaire consists of 45 items responded to on a 5-point Likert scale, where 1 = “very inapplicable” and 5 = “very applicable.” The 45th item, however, is unique to the German version of the questionnaire. Consequently, that item was omitted from the present study so that our results would be more comparable to those studies using other-language versions of the questionnaire. Mean scores were calculated for each Big Five trait, whose respective scales evidenced the following internal-consistency estimates (Cronbach’s alphas) in the present study: 0.76 for Openness; 0.76 for Conscientiousness; 0.83 for Extraversion; 0.67 for Agreeableness; and 0.82 for Neuroticism. Despite the possibility to calculate facet scores with the BFI, we do not present these in the current work.

#### Trust Propensity

TP was assessed by the German version of the disposition-to-trust scale, as presented in Gefen (2000), translated to German by a forward-and-backward translation procedure. The scale comprises five items responded to on a 7-point Likert scale, where 1 = “strongly disagree” and 7 = “strongly agree.” A mean score was computed; the Cronbach’s alpha of TP in the present study was 0.87.

#### E-Vendor Trust

Three items similar to those used by Gefen (2000) were applied to assess EVT; each item was translated to German by a forward-and-backward translation procedure. The items are responded to on a 7-point Likert scale, where 1 = “strongly disagree” and 7 = “strongly agree.” A mean score across all three items was computed; the Cronbach’s alpha of EVT in the present study was 0.93.

#### Need for Touch

NFT was assessed by the German version of the NFT scale (Nuszbaum et al., 2010) originally published by Peck (1999). The scale includes a total of 12 items—six assessing Autotelic NFT and six assessing Instrumental NFT. All items are responded to on a 7-point Likert scale, where 1 = “not at all true” to 7 = “exactly true.” Mean scores were calculated for Autotelic NFT and Instrumental NFT, whose Cronbach’s alphas were 0.94 and 0.93, respectively.

#### Willingness to Purchase Online/In-Store

Willingness to purchase online and willingness to purchase in-store were each assessed by two items similar to those set forth in McElroy et al. (2007); all items were translated to German by a forward-and-backward translation procedure. Once again, item responses were issued on a Likert scale, where 1 = “very inapplicable” and 5 = “very applicable.” The Spearman item correlation for the two items assessing willingness to purchase online was rho = 0.69, *p* < 0.001; for the two items assessing willingness to purchase in-store, it was rho = 0.70, *p* < 0.001 (rho, rather than Cronbach’s alpha, is presented here because the scales comprise just two items each).

### Statistical Analysis

The software package IBM SPSS Statistics Version 26 was used to perform the statistical analysis.

#### Analysis

The skewness and kurtosis of all scales were inspected for the survey sample as a whole and for the male and female subsets, respectively. Willingness to purchase products in-store showed a skewness (−2.31) and kurtosis (6.56) exceeding ±1, and the TP and EVT scales also showed a kurtosis exceeding ±1 in the total sample. In men and women, especially the scale to assess willingness to purchase products in-store showed a skewness and kurtosis exceeding ±1; next to skewness and kurtosis of some other variables, which just exceeded 1. According to the criteria by Miles and Shevlin (2001) but also taking into account the large sample size, we deemed especially the scale to assess willingness to buy products in-store as problematic and decided to use non-parametric tests whenever this scale was investigated.

Descriptive statistics and gender differences, as well as associations with age, were calculated using *t*-tests (Welch’s *t*-tests, whenever necessary) or Mann–Whitney *U* Tests and Pearson or Spearman correlations. To investigate differences between individuals with varying educational attainment, education was dummy coded, with 0 = “no kind of university degree” and 1 = “some kind of university degree”; this new grouping was necessary because the original groups were decidedly unbalanced in size. These two groups were compared by means of *t*-tests (Welch’s *t*-tests whenever necessary) or Mann–Whitney *U* Tests.

Next, zero-order bivariate correlations were calculated to investigate associations between study variables and determine which variables would be included in the final regression analysis.

Finally, to determine how much variance in willingness to purchase online and willingness to purchase in-store was explained by the Big Five, trust, and NFT, we performed hierarchical regression analyses. Accordingly, those variables that were significantly correlated with the putative dependent variables were entered in blocks. To predict willingness to purchase online, we entered the variables into the models as follows:

- Block 1: control variables (age, gender, and education)
- Block 2: trust variables (TP and EVT)
- Block 3: NFT (Instrumental NFT and Autotelic NFT)
- Model 1: Block 1 variables
- Model 2: Block 1 + Block 2 variables
- Model 3: Block 1 + Block 2 + Block 3 variables

Big Five traits were not included in these models because their correlations with willingness to purchase online were insignificant (see section “Results” below).

To predict willingness to purchase in-store, we entered the variables into the models as follows:

- Block 1: control variables (age, gender, and education)
- Block 2: all Big Five traits (Openness, Conscientiousness, Extraversion, Agreeableness, and Neuroticism)
- Block 3: trust variables (TP and EVT)
- Block 4: NFT (Instrumental NFT)
- Model 1: Block 1 variables
- Model 2: Block 1 + Block 2 variables
- Model 3: Block 1 + Block 2 + Block 3 variables
- Model 4: Block 1 + Block 2 + Block 3 + Block 4 variables

Autotelic NFT was not included in these models because the correlation with willingness to purchase in-store was not significant (see section “Results” below). Preliminary analyses were conducted to detect outliers and to check homoscedasticity. Outliers for each model were detected *via* Cook’s Distance values. There was, however, no case with a Cook’s Distance above the cut-off value of 1. The visual inspection of scatter plots further substantiated that homoscedasticity was not violated.

## Results

### Descriptive Statistics and Associations With Gender, Age, and Educational Attainment

Descriptive statistics for the total sample, and for men alone and women alone, as well as comparisons of men and women, can be found in the Appendix Table A1. Our findings in this realm include that women score significantly higher than men in Conscientiousness, Extraversion, Agreeableness, Neuroticism, Autotelic NFT, and Instrumental NFT, as well as in willingness to purchase products both online and in-store; effect sizes for these findings were small to medium (Cohen, 1988, 1992).

Age is significantly related to Openness (*r* = 0.07, *p* = 0.019), Conscientiousness (*r* = 0.06, *p* = 0.033), Neuroticism (*r* = −0.10, *p* < 0.001), Autotelic (*r* = −0.08, *p* = 0.007) and Instrumental (*r* = −0.07, *p* = 0.023) NFT, and willingness to purchase products online (*r* = −0.23, *p* < 0.001) and in-store (rho = −0.07, *p* = 0.014).

Differences in the two groups with different educational backgrounds were observed in Openness [*t*(1206) = −6.96, *p* < 0.001, *D* = 0.41; higher in individuals with some kind of university degree], Extraversion [*t*(1206) = −2.30, *p* = 0.022, *D* = 0.14; higher in individuals with some kind of university degree], EVT [*t*(901.5) = 3.33, *p* < 0.001, *D* = 0.20; higher in individuals without university degree], and Autotelic NFT [*t*(1206) = −2.43, *p* = 0.015, *D* = 0.14; higher in individuals with some kind of university degree].

These results support the assumption that the effects of gender, age, and educational background should be controlled in the final regression analyses.

### Zero-Order Bivariate Correlations

Table 2 lists zero-order bivariate correlations between all study variables of interest. Regarding associations between putative independent variables and dependent variables in the final models, the willingness to purchase online was significantly positively correlated with TP and EVT, but significantly negatively correlated with Autotelic NFT and Instrumental NFT, as the table shows. Openness, Conscientiousness, Extraversion, Agreeableness, TP, EVT, and Instrumental NFT were all positively significantly associated with willingness to purchase in-store, while Neuroticism was significantly negatively associated with it.

**Table 2:** Zero-order bivariate correlations between study variables in the total sample.

		1.	2.	3.	4.	5.	6.	7.	8.	9.	10.
1.	Openness										
2.	Conscientiousness	*r* = 0.20, *p* < 0.001									
3.	Extraversion	*r* = 0.32, *p* < 0.001	*r* = 0.37, *p* < 0.001								
4.	Agreeableness	*r* = 0.20, *p* < 0.001	*r* = 0.28, *p* < 0.001	*r* = 0.19, *p* < 0.001							
5.	Neuroticism	*r* = −0.26, *p* < 0.001	*r* = −0.34, *p* < 0.001	*r* = −0.34, *p* < 0.001	*r* = −0.34, *p* < 0.001						
6.	Trust Propensity	*r* = 0.14, *p* < 0.001	*r* = 0.15, *p* < 0.001	*r* = 0.22, *p* < 0.001	*r* = 0.48, *p* < 0.001	*r* = −0.20, *p* < 0.001					
7.	E-Vendor Trust	*r* = 0.02, *p* = 0.541	*r* = 0.15, *p* < 0.001	*r* = 0.14, *p* < 0.001	*r* = 0.19, *p* < 0.001	*r* = −0.11, *p* < 0.001	*r* = 0.31, *p* < 0.001				
8.	Autotelic Need for Touch	*r* = 0.03, *p* = 0.276	*r* = −0.16, *p* < 0.001	*r* = 0.02, *p* = 0.443	*r* = −0.02, *p* = 0.445	*r* = 0.17, *p* < 0.001	*r* = 0.05, *p* = 0.074	*r* = −0.09, *p* = 0.002			
9.	Instrumental Need for Touch	*r* = 0.03, *p* = 0.296	*r* = −0.03, *p* = 0.269	*r* = 0.01, *p* = 0.823	*r* = 0.04, *p* = 0.194	*r* = 0.07, *p* = 0.013	*r* = 0.13, *p* < 0.001	*r* = −0.03, *p* = 0.267	*r* = 0.67, *p* < 0.001		
10.	Willingness to Purchase Online	*r* = 0.02, *p* = 0.529	*r* = 0.03, *p* = 0.299	*r* = 0.03, *p* = 0.297	*r* = 0.04, *p* = 0.137	*r* = 0.01, *p* = 0.638	*r* = 0.07, *p* = 0.019	*r* = 0.34, *p* < 0.001	*r* = −0.08, *p* = 0.005	*r* = −0.14, *p* < 0.001	
11.	Willingness to Purchase In-Store	rho = 0.10, *p* < 0.001	rho = 0.16, *p* < 0.001	rho = 0.16, *p* < 0.001	rho = 0.15, *p* < 0.001	rho = −0.07, *p* = 0.018	rho = 0.19, *p* < 0.001	rho = 0.32, *p* < 0.001	rho = 0.01, *p* = 0.743	rho = 0.13, *p* < 0.001	rho = 0.32, *p* < 0.001

All results are based on the total sample (*N* = 1,208). Values of *p* are not corrected for multiple testing, because these correlational analyses served only to determine which variables would be included in the final hierarchical regression models. However, a manual check of the 19 correlations (see row 10. and 11.) that respond to the putative dependent variables willingness to purchase online and willingness to purchase in-store revealed that nearly all significant correlations survived the Bonferroni–Holm correction, the sole exception being the associations between willingness to purchase in-store and Neuroticism (0.018 ^*^ 8 = 0.144) as well as willingness to purchase online and Trust Propensity (0.019 ^*^ 7 = 0.133). A manual check of all 55 correlations revealed that the significant associations between willingness to purchase online and Autotelic NFT (0.005 ^*^ 20 = 0.100), Neuroticism and Instrumental NFT (0.013 ^*^ 19 = 0.247), willingness to purchase in-store and Neuroticism (0.018 ^*^ 18 = 0.324), as well as willingness to purchase online and Trust Propensity (0.019 ^*^ 17 = 0.323) did not survive the Bonferroni–Holm correction.

In light of the hierarchical, blockwise regressions, we entered those variables that were significantly correlated with the putative dependent variable, as per the method described above in the section “Materials and Methods”.

Tables 3 and 4 report the results.

**Table 3:** Results of the hierarchical regression models predicting willingness to purchase online.

	Model 1	Model 2	Model 3
**Block 1**			
Age	−0.23^⁎⁎⁎^	−0.23^⁎⁎⁎^	−0.23^⁎⁎⁎^
Gender	0.02	0.02	0.04
Education	−0.00	0.03	0.04
**Block 2**			
Trust Propensity		−0.03	−0.01
E-Vendor Trust		0.36^⁎⁎⁎^	0.35^⁎⁎⁎^
**Block 3**			
Autotelic NFT			0.05
Instrumental NFT			−0.18^⁎⁎⁎^
**Summary Statistics**			
*F*	23.63^⁎⁎⁎^	52.17^⁎⁎⁎^	43.26^⁎⁎⁎^
F Change	23.63^⁎⁎⁎^	89.75^⁎⁎⁎^	17.40^⁎⁎⁎^
Change in *R*^2^	0.06	0.12	0.02
Adjusted *R*^2^	0.05	0.18	0.20

*Due to statistical reasons, only men and women were included in this model, while individuals stating “third gender or gender-diverse” were excluded. Statistics are beta values. Change in *R*^2^ is based on *R*^2^ and not Adjusted *R*^2^*.

⁎⁎⁎ *p < 0.001*.

**Table 4:** Results of the hierarchical regression models predicting willingness to purchase in-store.

	Model 1	Model 2	Model 3	Model 4
**Block 1**				
Age	−0.06	−0.08^⁎⁎^	−0.07^⁎^	−0.07^⁎^
Gender	0.04	0.01	0.02	0.01
Education	0.04	0.02	0.04	0.04
**Block 2**				
Openness		0.06	0.07^⁎^	0.06^⁎^
Conscientiousness		0.06^⁎^	0.05	0.06
Extraversion		0.06^⁎^	0.03	0.03
Agreeableness		0.09^⁎⁎^	0.02	0.02
Neuroticism		0.03	0.04	0.03
**Block 3**				
Trust Propensity			0.08^⁎⁎^	0.07^⁎^
E-Vendor Trust			0.22^⁎⁎⁎^	0.23^⁎⁎⁎^
**Block 4**				
Instrumental NFT				0.08^⁎⁎^
**Summary Statistics**				
*F*	3.29^⁎⁎^	5.67^⁎⁎⁎^	12.66^⁎⁎⁎^	12.40^⁎⁎⁎^
*F* Change	3.29^⁎⁎^	7.05^⁎⁎⁎^	39.17^⁎⁎⁎^	8.97^⁎⁎^
Change in *R*^2^	0.01	0.03	0.06	0.01
Adjusted *R*^2^	0.01	0.03	0.09	0.09

*Due to statistical reasons, only men and women were included in this model, while individuals stating “third gender or gender-diverse” were excluded. Statistics are beta values. Change in *R*^2^ is based on *R*^2^ and not Adjusted *R*^2^*.

⁎⁎⁎ *p* *< 0.001;*

⁎⁎ *p* *< 0.01;*

⁎ *p < 0.05*.

### Research Objective 1: The Big Five and Willingness to Purchase Online and In-Store

As demonstrated in the zero-order bivariate correlations, all Big Five traits are uncorrelated with willingness to purchase online (see Table 2). Consequently, the Big Five were not included in the hierarchical regression models to predict customers’ willingness to purchase online. When examining willingness to purchase in-store, however, the inclusion of the Big Five explains incremental variance after age, gender, and education have been considered in the analysis (Table 4, Model 2). More specifically, willingness to purchase in-store is significantly positively related to Extraversion (*β* = 0.06, *p* = 0.048), Agreeableness (*β* = 0.09, *p* = 0.004), and Conscientiousness (*β* = 0.06, *p* = 0.046). No associations were found between willingness to purchase in-store and Neuroticism or Openness in Model 2. However, when the trust variables (Table 4, Model 3) and Instrumental NFT (Table 4, Model 4) are added to the regressions, Openness (*β* = 0.07, *p* = 0.030, Model 3; *β* = 0.06, *p* = 0.039, Model 4) becomes the only Big Five trait to display a significant and positive correlation with willingness to purchase in-store.

### Research Objective 2: Trust and Willingness to Purchase Online and In-Store

As can be seen in Table 3, trust variables explain incremental variance in willingness to purchase online after age, gender, and educational attainment have been considered in the analysis. Specifically, although TP is not significantly associated with willingness to purchase online, EVT (*β* = 0.36, *p* < 0.001, Model 2; *β* = 0.35, *p* < 0.001, Model 3) predicts willingness to purchase online significantly. For willingness to purchase in-store, trust variables explain incremental variance after age, gender, education, and the Big Five have been considered in the analysis (Table 4). Consequently, TP (*β* = 0.08, *p* = 0.011, Model 3; *β* = 0.07, *p* = 0.034, model 4) and EVT (*β* = 0.22, *p* < 0.001, Model 3; *β* = 0.23, *p* < 0.001, Model 4) both significantly positively predict customer willingness to purchase in-store.

### Research Objective 3: Need for Touch and Willingness to Purchase Online and In-Store

NFT explains incremental variance in willingness to purchase online after age, gender, education, and trust have been considered in the analysis (Table 3, Model 3). While Autotelic NFT is not significantly associated, especially Instrumental NFT (*β* = −0.18, *p* < 0.001) is negatively associated with the willingness to purchase online. Moreover, Instrumental NFT also explains incremental variance in willingness to purchase in-store after age, gender, education, the Big Five, and trust have been considered in the analysis (Table 4, Model 4). Instrumental NFT (*β* = 0.08, *p* = 0.003) is positively associated with willingness to purchase in-store.

## General Discussion

With the notable exception of Breazeale and Lueg (2011), most research on the influence of any of TP, EVT, and Big Five traits on customers willingness to purchase has considered just one retail channel, which is typically online. Indeed, to the best of our knowledge, no study has considered the effects of personality constructs (such as the Big Five) alongside the customer’s levels of TP, EVT, and NFT in the context of both online and in-store shopping (Hermes and Riedl, 2021b). Given the paucity of academic literature on these constructs in simultaneous relation to online and in-store purchasing channels, this study addresses a significant research gap.

The aim of the present study was to explore how the customers’ willingness to purchase in-store and online is related to the Big Five personality traits (Research Objective 1), the trust variables TP and EVT (Research Objective 2), and Instrumental and Autotelic NFT (Research Objective 3). Regarding our first objective, the findings of this study reveal that the Big Five explained a significant amount of variance in willingness to purchase in-store but could not explain variance in willingness to purchase online. Pursuing our second objective, we found that TP was significantly, positively associated with willingness to purchase in-store, but not with willingness to purchase online, while EVT significantly predicted both in-store and online purchase willingness. As for our final objective, Instrumental NFT demonstrated significant capability to explain both willingness to purchase in-store (positively) and willingness to purchase online (negatively).

### Research Objective 1: The Big Five and Willingness to Purchase Online and In-Store

The Big Five predicted willingness to purchase in-store but not willingness to purchase online; this result partially supports Proposition 1. Model 2 (Table 4) demonstrates that Extraversion, Agreeableness, and Conscientiousness are positively (and significantly) correlated with willingness to purchase in-store, whereas Neuroticism and Openness are not. A review of past literature can help to explain this finding: individuals high in Extraversion are socially active (Costa and McCrae, 1992) and have been found to prefer shopping in malls compared to shopping online (Breazeale and Lueg, 2011). Hence, it should come as no surprise that individuals high in Extraversion are more likely to prefer in-store shopping compared to individuals scoring lower in Extraversion. Further, people high in Agreeableness are characterized by interpersonal and cooperative behavior (Costa and McCrae, 1992). When shopping in-store, there are likely other customers or sales personnel present, resulting in a social environment that allows for interpersonal and cooperating behavior (Verhoef et al., 2009). Hence, the previously mentioned characteristics of people high in Agreeableness help explain their higher willingness to purchase in-store. Lastly, individuals high in Conscientiousness tend to plan ahead and are well organized (Costa and McCrae, 1992), and shop for utilitarian reasons (Guido, 2006). Along these lines, our findings suggest that those customers’ shopping needs can be better fulfilled when shopping in-store (e.g., a visit to the shop is better plannable than to wait for an online delivery which might be missed or delayed). Of note, however, is that after incorporating the two trust variables (TP and EVT) and after incorporating Instrumental NFT, Openness was the sole significant predictor of in-store purchase willingness remaining (Table 4, Models 3 and 4), highlighting the overarching role of trust, but also the crucial role of Instrumental NFT, in predicting willingness to purchase in-store.

Surprisingly, however, the Big Five failed to explain willingness to purchase online. The question therefore arises: why do the Big Five explain in-store, but not online, willingness to purchase? Studies in a variety of countries, including Croatia (Bosnjak et al., 2007), the United States (Breazeale and Lueg, 2011), Malaysia (Mohamed et al., 2014), and Taiwan (Moslehpour et al., 2018), have found that some of the Big Five traits can predict online shopping behavior, yet a study of German-speaking participants, which is also the population of this study, failed to replicate this conclusion Piroth et al., (2020a). The market structures of Germany and Austria are characterized by a high density of shops like supermarkets (Ifh/HDE e. V., 2017; Wollenburg et al., 2018) and lower rates of e-commerce sales (in comparison with, e.g., the United States; Mastercard Economics Institute/Statista, 2021). Further, most German-speaking retailers have been found to lack a compelling online service and communication strategy, making their services less digitalized and personalized (Piroth et al., 2020b). In contrast, it is possible—especially in physical stores—for sales personnel to adjust their behavior to expressions of customers’ personalities (e.g., anxiety; Spiro and Weitz, 1990; Esmark and Noble, 2018), potentially creating advantageous matches between customers and salespeople and possibly resulting in highly personalized in-store experiences (Crosby et al., 1990; Dwyer et al., 1998; Prendergast et al., 2014; Hall et al., 2015). Hence, while more research is needed on the role of a customer’s personality and on the sales personnel’s and website’s ability to adapt to such, we believe that different levels of personalization constitute a possible explanation for our findings.

### Research Objective 2: Trust and Willingness to Purchase Online and In-Store

Both EVT and TP were positively associated with willingness to purchase in-store, and EVT was positively associated with willingness to purchase online; this result partially supports Proposition 2. Specifically, that both EVT and TP were positively associated with in-store purchase willingness supports our reasoning that trust can flow from the online to the offline channel, particularly, trusting an e-vendor has a positive effect on making purchases in that vendor’s physical stores as well. This is an important finding as it demonstrates that retailers’ investments in their online shops might result in positive spillover effects that can also affect their physical stores. We are not aware of published research providing evidence that especially EVT has this effect within the domain of fashion retailing.

EVT was also an especially strong positive predictor of willingness to purchase online. This affirms previous studies that identified positive correlations between EVT and online shopping intentions (Gefen and Straub, 2003; Gefen et al., 2003a; Kim, 2014), use of e-vendor websites (Gefen et al., 2003b; Kim, 2008), and willingness to commit in a long-term relationship with the retailer (Cho, 2006). However, TP was not associated with willingness to purchase online, contradicting our proposal. Because the previously published findings regarding TP’s effect on willingness to purchase online are inconclusive—Bianchi and Andrews (2012), for example, found a negative effect, while McKnight et al. (2002b) found no effect at all—this study concludes that TP seems to play only a minor and insignificant role in predicting customers’ willingness to purchase online. At the very least, our data suggest scholars should cautiously avoid overestimating a possible effect of this type.

Lastly, considering the online and in-store purchasing associations, we found evidence that the effect of EVT on willingness to purchase online is stronger than those of Instrumental NFT; and the combined effect of both TP and EVT on willingness to purchase in-store is stronger than the effect of the Big Five and the Instrumental NFT. Thus, our findings mirror the existing literature when it comes to recognizing the importance of trust, both in marketing at large (Morgan and Hunt, 1994; Heidarian, 2019) and for online shoppers in particular (McKnight et al., 1998; Gefen, 2000). Finally, we note that as online shopping is considered risky partly because of the absence of haptic product information (Peck and Childers, 2003a,b), it is unsurprising that a customer’s level of trust in a given e-vendor should prove more important than factors, such as NFT.

### Research Objective 3: Need for Touch and Willingness to Purchase Online and In-Store

Instrumental NFT, but not Autotelic NFT, had a significant impact on both in-store and online willingness to purchase, affecting the former positively and the latter negatively. These results partially support Proposition 3. The identification of a positive relationship between Instrumental NFT and in-store purchase willingness joins previous studies in demonstrating that people with high levels of NFT prefer to buy products through retail channels that allow for touch, such as physical stores (Peck and Childers, 2006; Cho and Workman, 2011; Workman and Cho, 2013; Shankar and Jain, 2021). Correspondingly, Instrumental NFT was negatively associated with willingness to purchase online. This finding, too, is in agreement with published literature: Kühn et al. (2020) found that customers with high NFT experience stronger quality concerns when shopping online, and San-Martín et al. (2017) found that customers scoring lower in NFT were more strongly oriented toward e-commerce (although Duarte and e Silva (2020) found no association between NFT and propensity to make online purchases). Overall, our findings can be explained by the lack of haptic product information available to online customers (Citrin et al., 2003), which makes it more difficult for customers with high NFT to evaluate a product’s quality (Kühn et al., 2020). Because we know that emotions play a crucial role in the customer experience (Gentile et al., 2007; Puccinelli et al., 2009; Hermes and Riedl, 2020a,b, 2021a), it is surprising that Autotelic NFT did not evince a significant association with either online or in-store willingness to purchase. However, it must be observed that the study data were collected in the midst of the COVID-19 pandemic, during which a notable number of customers have perceived in-store shopping as vector of infection and took corresponding precautions that might disrupt or mitigate Autotelic NFT (e.g., disinfecting their hands; Szymkowiak et al., 2021). Additionally, customers’ product-touching habits themselves have changed (Willems et al., 2021). Hence, we believe that customers’ fear of contracting COVID-19 might have resulted in lower levels of Autotelic NFT, and possibly also lower levels of Instrumental NFT.

### Theoretical and Practical Implications

The online- and in-store-purchase associations found in the present work contribute to the literature in several ways. First, the research investigates potential drivers of in-store and online willingness to purchase, which the ascent of contemporary omnichannel retailing has made increasingly important. The findings of this study therefore both supplement existing knowledge and open new doors for future research. The study considers the predictive power of customers’ personality, levels of trust, and levels of NFT on their willingness to purchase, both in-store and online. In response to the rise of e-commerce during the COVID-19 pandemic (Scott et al., 2020; Sheth, 2020), this research offers insights into which retail channels are preferred by various kinds of customers, as well as where those preferences might originate. Second, we discover that the Big Five predict willingness to purchase in-store but not willingness to purchase online, and we propose explanations for this phenomenon. This novel distinction represents an insight added to Big Five personality literature. Third, we demonstrate how trust is a strong predictor of both in-store and online willingness to purchase, substantiating findings made by previous researchers. Consequently, we find that while EVT is related to willingness to purchase through either channel, TP is only related to willingness to purchase in-store. Lee et al. (2014) identified a spillover effect of trust from an online website to an “offline site” (e.g., an e-vendor’s link to information about a physical store location) in a cosmetic retailing context. However, our study is the first one to empirically demonstrate the links of both EVT and TP on in-store shopping behavior for fashion customers, also providing evidence for a positive spillover effect from EVT to in-store purchase willingness. Fourth, the role of NFT, as well as those of its subconstructs, Instrumental NFT and Autotelic NFT, are well explained by the associations we identify between NFT and in-store and online purchasing. More specifically, Instrumental NFT alone is positively associated with willingness to purchase in-store and negatively associated with willingness to purchase online.

The shopping experience is unceasingly created through the interplay of retail touchpoints and the customer’s reactions to them (Verhoef et al., 2009). These reactions, in turn, might be strongly influenced by factors, such as the Big Five, trust, and NFT. Therefore, it is critical in both theory and practice to focus on customers’ inherent, stable characteristics (Gentile et al., 2007; Puccinelli et al., 2009; Verhoef et al., 2009). Importantly, retailers today typically have access to substantial pools of customer data, which can be taken advantage of to personalize and optimize services (Zhang and Sundar, 2019; Razavi, 2020; Stachl et al., 2020). Accordingly, this study demonstrates the necessity that retailers look beyond mere demographic data on consumers. For example, the knowledge that high Agreeableness, high Conscientiousness, and high Extraversion in customers was associated with the willingness to purchase in-store (as long as other variables such as NFT and trust were not included in the model) might allow retailers to train their in-store sales personnel on how best to serve these personalities in particular.

Other knowledge from this research can be similarly applied in practice. For example, EVT was a significant predictor of both online and in-store purchase willingness, while TP was positively and significantly associated only with willingness to purchase in-store. Though retailers may not be able to exercise influence on customers’ levels of TP, they can still implement strategies to increase EVT, such as giving customers access to a privacy dashboard through which they could control the personal data collected by the retailer (Herder and Van Maaren, 2020). Because EVT was positively associated with willingness to purchase in-store just as well as willingness to do so online, strategically cultivating EVT among consumers could prove to be a critical strategy. Lastly, Instrumental NFT was positively associated with the willingness to purchase in-store but showed a negative association with the willingness to purchase online. This finding should encourage retailers to support in-store customers in touching products, but it should also inform retailers that offering a sufficiently clean and safe environment during the COVID-19 pandemic and beyond is crucial. For customers with low levels of Instrumental NFT, one implication could be that retailers could offer innovative technologies (e.g., virtual or augmented reality, VR, or AR) to provide them with product information prior to purchase. The fact that some people do not need to touch products prior purchase does not necessarily mean that they do not need, or prefer, information at all. Thus, information could be provided *via* optical and acoustic senses, such as afforded by VR and AR applications (rather than *via* haptic senses).

### Limitations and Future Research

This exploratory study expands the existing knowledge regarding the associations of the Big Five, trust, and NFT with in-store and online willingness to purchase. However, the work is bound by certain limitations, one of which is its limited scope. This study focused on online and in-store shopping channels in fashion retailing, the industry from which the survey participants were drawn. This specific focus has implications for the generalizability of these isolated results. To determine whether they hold true in other industries or consider the variables’ relations to other channels, future research should replicate the study in other product categories, other purchasing channels (e.g., augmented/virtual reality interfaces; Lixăndroiu et al., 2021; Mishra et al., 2021), or both. Another challenge is that the sample examined in the present study only includes German-speaking individuals. Future research could work to validate our findings in different geographical areas and, thus, collect samples representing different socio-cultural backgrounds.

Valuable insights might also be generated by altering the variables used in this study. Although we considered the relations of the Big Five with online and in-store willingness to purchase, other models of personality exist and could be examined. Future studies might gain additional knowledge by considering, for example, personality theories, such as the one basing on Affective Neuroscience theory (Davis et al., 2003; Montag and Davis, 2018). Another rewarding avenue of future research might be to determine the role of customers’ social experiences in purchase-channel selection—e.g., how does the ability of sales personnel to adjust to customers’ personalities affect purchase-channel selection?

Additionally, future research might also turn up new findings by leaving some variables unchanged. In the current study, Autotelic NFT failed to predict either willingness to purchase online or willingness to purchase in-store. However, as outlined above, this finding may have been related to the fact that data collection took place during the COVID-19 pandemic. Hence, to establish the stability of the results of the present study, we recommend replicating this study post-pandemic. In contrast, TP was surprisingly a significant predictor of willingness to purchase in-store but not of willingness to purchase online. Future research could examine the influence of TP on specific online and in-store trust-building mechanisms—e.g., might customers with high TP be quicker to trust in-store staff, or will online trust-building strategies better work for these customers?

To once again touch on the limitations of this work, it must be noted that our findings are based on data gathered from self-reported questionnaires, so they may reflect biases in respondents’ judgment (Hufnagel and Conca, 1994). We also note that correlations are at maximum in the moderate size area; for instance, EVT and willingness to purchase in-store correlate at 0.32, meaning they share about 10% variance (0.32 * 0.32). Thus, many other variables must be taken into account to understand online and in-store willingness to purchase. Lastly, the present research is of correlational nature; therefore, no causality can be inferred from our findings. To understand causal links between the explored variables, longitudinal or experimental work is required.

## Conclusion

Research incorporating multiple retail channels is of critical importance, especially in today’s omnichannel environment. Prior research has revealed that customers’ purchase-channel decisions may be affected by their personality, level of trust, and level of NFT. However, such research almost never considered the relations of these factors across multiple retail channels. Against this background, we conducted a survey study with a large sample (*N* = 1,208) to explore how the Big Five, trust, and NFT were related to willingness to purchase across two retail channels, in-store and online. We found that Extraversion, Agreeableness, and Conscientiousness predicted willingness to purchase in-store (though it should be noted that after the introduction of TP and EVT, as well as after the introduction of Instrumental NFT, to the regression model, Openness was the only Big Five trait being significantly and positively correlated with in-store purchase willingness). TP, EVT, and Instrumental NFT were all found to be positively related to willingness to purchase in-store, while there was no association with Autotelic NFT. Moreover, EVT was positively, and Instrumental NFT negatively, associated with willingness to purchase online at a significant level; none of the Big Five traits, nor TP or Autotelic NFT, had such an association in the regression model. Since this study was conducted during the COVID-19 pandemic, we call for future research to replicate our exploratory findings post-pandemic. Such replication would shed further light on the important, yet under-researched, domain of psychological drivers of purchase behavior across offline and online retail environments.

## Data Availability Statement

The datasets presented in this article are not readily available because of a Confidentiality Agreement. Requests to access the datasets should be directed to anna.hermes@jku.at.

## Ethics Statement

Ethical review and approval was not required for the study on human participants in accordance with the local legislation and institutional requirements. The participants provided their electronic informed consent to participate in this study.

## Author Contributions

RR, CM, CS, and AH: conceptualization and methodology. CS and AH: data curation, formal analysis, validation, and visualization. RR: funding acquisition. AH: project administration. AH and RR: writing—original draft. CS and CM: writing—review and editing. All authors have read and agreed to the published version of the manuscript.

## Funding

This study has been conducted within the training network project PERFORM funded by the European Union’s Horizon 2020 research and innovation program under the Marie Skłodowska-Curie grant agreement no. 765395.

## Conflict of Interest

The authors declare that the research was conducted in the absence of any commercial or financial relationships that could be construed as a potential conflict of interest.

## Publisher’s Note

All claims expressed in this article are solely those of the authors and do not necessarily represent those of their affiliated organizations, or those of the publisher, the editors and the reviewers. Any product that may be evaluated in this article, or claim that may be made by its manufacturer, is not guaranteed or endorsed by the publisher.

## Figures and Tables

**Table A1:** Descriptive statistics and gender differences.

	Total Sample (*N* = 1,208)	Men (*n* = 596)	Women (*n* = 609)	Gender Differences	Effect Size
	*M* (*SD*)	*M* (*SD*)	*M* (*SD*)		
Openness	3.43 (0.55)	3.46 (0.54)	3.41 (0.57)	*t*(1203) = 1.37, *p* = 0.172	*D* = 0.08
Conscientiousness	4.09 (0.51)	4.04 (0.53)	4.14 (0.49)	*t*(1203) = −3.54, *p* < 0.001	*D* = 0.20
Extraversion	3.68 (0.68)	3.62 (0.68)	3.74 (0.66)	*t*(1203) = −3.09, *p* = 0.002	*D* = 0.18
Agreeableness	3.67 (0.49)	3.61 (0.49)	3.73 (0.49)	*t*(1203) = −4.12, *p* < 0.001	*D* = 0.24
Neuroticism	2.40 (0.65)	2.28 (0.61)	2.51 (0.67)	*t*(1197.1) = −6.23, *p* < 0.001	*D* = 0.36
Trust Propensity	5.22 (0.99)	5.20 (0.96)	5.26 (1.00)	*t*(1203) = −1.08, *p* = 0.281	*D* = 0.06
E-Vendor Trust	5.76 (0.97)	5.76 (0.95)	5.78 (0.97)	*t*(1203) = −0.45, *p* = 0.653	*D* = 0.03
Autotelic Need for Touch	3.09 (1.46)	2.85 (1.40)	3.33 (1.49)	*t*(1203) = −5.76, *p* < 0.001	*D* = 0.33
Instrumental Need for Touch	4.21 (1.49)	4.01 (1.54)	4.41 (1.43)	*t*(1192.6) = −4.72, *p* < 0.001	*D* = 0.27
Willingness to Purchase Products Online	3.87 (1.02)	3.79 (0.99)	3.94 (1.05)	*t*(1203) = −2.53, *p* = 0.011	*D* = 0.15
Willingness to Purchase Products In-Store	4.51 (0.78)	4.47 (0.76)	4.56 (0.79)	*W* = 163103, *p* < 0.001	*r* = −0.10

*Individuals stating “third gender or gender-diverse” as gender identity were not included in the comparison between genders due to the small number of individuals in this group. Mean gender differences in Neuroticism and Instrumental NFT were tested using Welch’s *t*-test (due to a significant Levene’s test)*.

## Footnotes

^1^This study was preregistered (https://osf.io/b4ejm). Due to current events, namely, the possible impact of the COVID-19 pandemic on customers’ spending behavior (Di Crosta et al., 2021) and touch preferences (Willems et al., 2021), the current study deviates from the preregistration (e.g., in the present work we do not specifically test the relationships between the Big Five traits and Autotelic and Instrumental NFT). However, all preregistered variables are included in the manuscript, and the corresponding zero-order bivariate correlations mentioned in the preregistration can be found in Table 2. Owing to a lack of sufficient data, we also used survey-only data in lieu of the preregistered combination of survey data and objective purchase data.

^2^ https://www.limesurvey.org/

## Appendix

### Appendix A

Appendix A1 Data Cleaning

None of the participants needed to be excluded due to missing data. However, *n* = 6 individuals indicated an implausibly old age. More specifically, all of them seem to have provided the year of their birth instead of their age. Because only the year was provided, no exact age could be determined, which is why these individuals were excluded. Next, *n* = 15 individuals were excluded because they chose the same response option throughout at least one of the survey’s pages on which Big Five Inventory items (see main manuscript) were presented indicating careless responding (this procedure to spot careless responding was only applied to the survey questions measuring the Big Five, this is because the Big Five measure is comparatively longer and includes recoded items which is not true for the other measures used in the present study).

## Abbreviations

TP: Trust Propensity
EVT: E-Vendor Trust
NFT: Need for Touch
